# Penile Foreskin Avulsion from Parrot Fish Bite

**DOI:** 10.5811/westjem.2015.1.25338

**Published:** 2015-02-26

**Authors:** Sow A. Kobayashi, Erica F. Sanford, Peter Witucki

**Affiliations:** * University of California, San Diego, Department of Emergency Medicine, La Jolla, California; † University of California, San Diego, Department of Pediatrics, La Jolla, California

A healthy, uncircumcised 34-year-old male presented to an emergency department (ED) in Tinian (Commonwealth of the Northern Mariana Islands) after a parrot fish bite. The patient was spearfishing in the Philippine Sea and impaled a 15-pound parrot fish. As the patient was attempting to grasp the speared fish it bit him in the groin exterior to his swimming trunks. He experienced immediate penile pain and swam back to shore. Upon removal of his intact swimming trunks, the patient noticed a two-centimeter foreskin avulsion at the proximal penile shaft (picture). He presented to the ED where extensive irrigation of the wound with normal saline was initiated, followed by administration of local anesthesia and laceration repair with sutures. The patient was offered systemic analgesics but declined. He received tetanus toxoid immunization and was discharged with an antibiotic prescription for skin and marine flora with instructions to follow up in one week. The patient was contacted 10 weeks later for follow-up and stated that the avulsion healed well with good comesis and he has had no infections or issues with urination or erections.

## Figures and Tables

**Figure:**
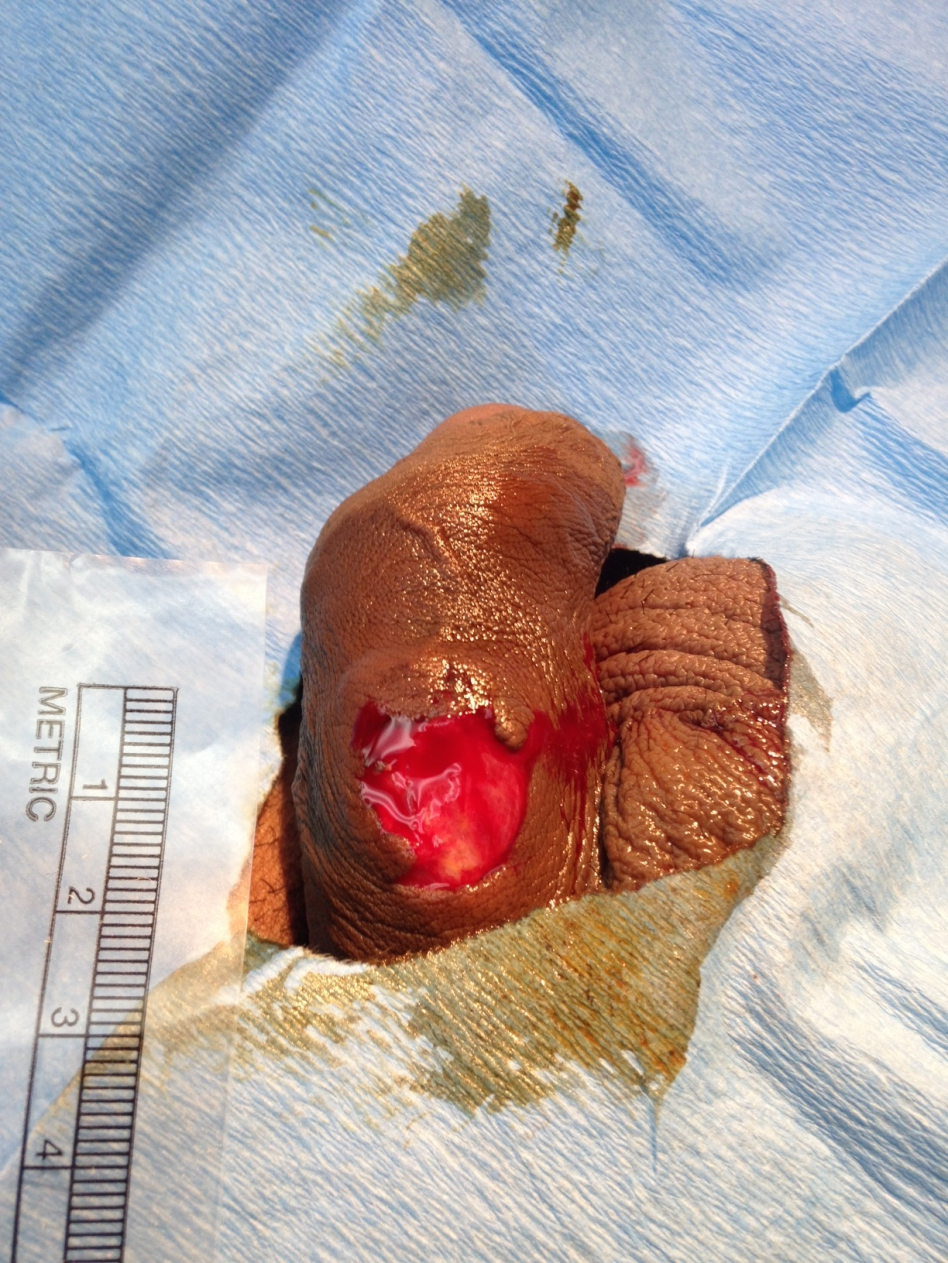
Penile foreskin avulsion from parrot fish bite.

